# Effects of Titanium Dioxide Nanoparticles Exposure on Human Health—a Review

**DOI:** 10.1007/s12011-019-01706-6

**Published:** 2019-04-13

**Authors:** Ewa Baranowska-Wójcik, Dominik Szwajgier, Patryk Oleszczuk, Anna Winiarska-Mieczan

**Affiliations:** 1 Department of Biotechnology, Microbiology and Human Nutrition, University of Life Sciences in Lublin, Skromna 8, 20-704 Lublin, Poland; 2 Department of Environmental Chemistry, Maria Curie-Skłodowska University in Lublin, Pl. M. Curie-Skłodowskiej 3, 20-031 Lublin, Poland; 3 Department of Bromatology and Food Physiology, University of Life Sciences in Lublin, Akademicka 13, 20-950 Lublin, Poland

**Keywords:** TiO_2_, Nanoparticles, Health, Food, Toxicity

## Abstract

Recently, an increased interest in nanotechnology applications can be observed in various fields (medicine, materials science, pharmacy, environmental protection, agriculture etc.). Due to an increasing scope of applications, the exposure of humans to nanoparticles (NPs) is inevitable. A number of studies revealed that after inhalation or oral exposure, NPs accumulate in, among other places, the lungs, alimentary tract, liver, heart, spleen, kidneys and cardiac muscle. In addition, they disturb glucose and lipid homeostasis in mice and rats. In a wide group of nanoparticles currently used on an industrial scale, titanium dioxide nanoparticles—TiO_2_ NPs—are particularly popular. Due to their white colour, TiO_2_ NPs are commonly used as a food additive (E 171). The possible risk to health after consuming food containing nanoparticles has been poorly explored but it is supposed that the toxicity of nanoparticles depends on their size, morphology, rate of migration and amount consumed. Scientific databases inform that TiO_2_ NPs can induce inflammation due to oxidative stress. They can also have a genotoxic effect leading to, among others, apoptosis or chromosomal instability. This paper gives a review of previous studies concerning the effects of exposure to TiO_2_ NPs on a living organism (human, animal). This information is necessary in order to demonstrate potential toxicity of inorganic nanoparticles on human health.

## Introduction

**Fig. 1:**
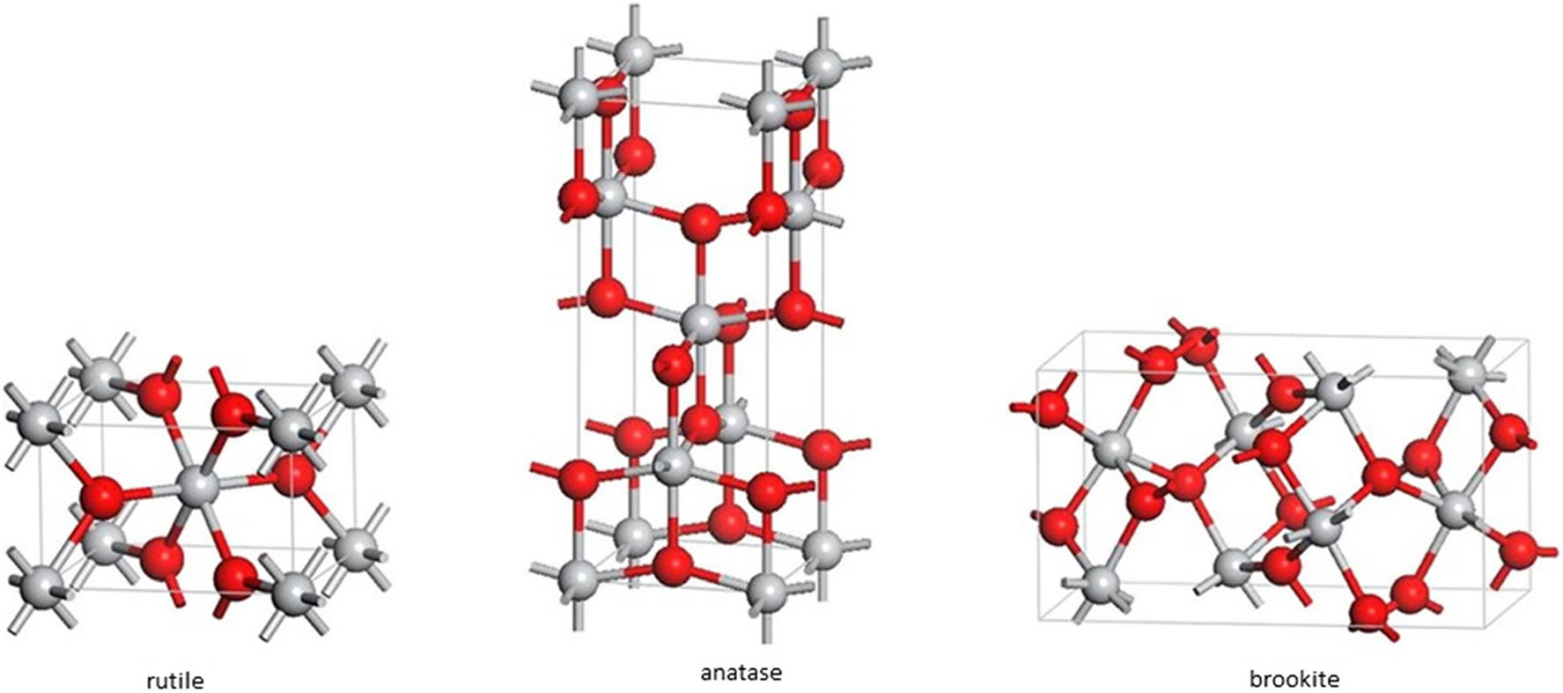
Tetragonal structures of crystalline forms of rutile, anatase and brookite TiO_2_ NPs (spheres: red—0_2_, grey—Ti). Based on Samat et al. [4]

Recently, nanotechnology has been a subject of great interest, offering considerable advantages in many areas. Titanium dioxide (TiO_2_ NPs) is among the most often used nanoparticles. The particle size depends on its application, including ultrafine particles < 100 nm, and fine particles 0.1 to ca. 3 μm [1]. It occurs in three different variants: as rutile, anatase and, more rarely, brookite [2, 3] (Fig. 1) [4]. With regard to its increased photocatalytic activity [3, 5], anatase in comparison to rutile and brookite has a higher number of industrial applications; however, it is the most toxic form [6].

## Occurrence of TiO_2_ NPs

**Fig. 2:**
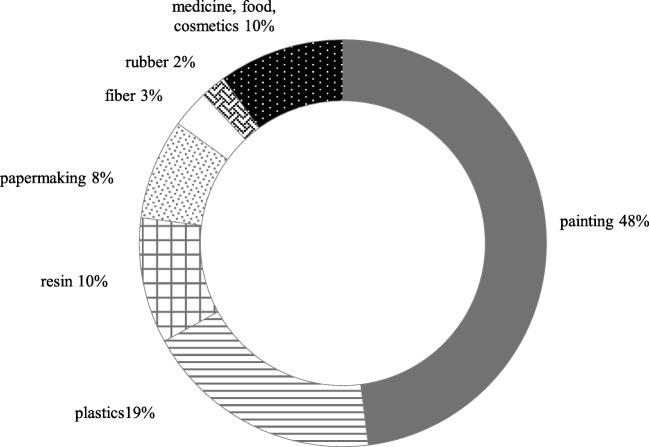
Application of TiO_2_ NPs (%) in industry. Based on Hong et.al [7]

TiO_2_ NPs is used in many areas of life, such as environmental protection and building engineering, medicine, agriculture and the food and cosmetic industry [4] (Fig. 2) [7]. With regard to its catalytic properties, TiO_2_ NPs are a component of self-cleaning roof tiles, windows, they are used in water and sewage treatment, gas combustion, as an antibacterial material for decontamination, as well as a catalyst in organic synthesis [2, 8]. Their biomedical applications include pharmaceuticals and medical devices [2]. In the agriculture industry, they are used in the production of fertilisers and pesticides which can significantly affect soil fertility, growth of plants and crop yield [9, 10]. TiO_2_ NPs have a wide range of applications in the food industry (E171) [11, 12]; they are used in the processing and packing of food for the purposes of product improvement. They are also used in the cosmetics industry, pharmaceuticals and toothpastes [13–15]. They have a wide-range antibacterial effect extending the shelf life of foodstuffs [16, 17]*.*

With regard to the fact that TiO_2_ NPs are used widely and commonly in many areas of industry, the risk of exposure increases; hence, their potential effect on the human body should be explored in more detail.

## TiO_2_ NPs in Foodstuffs

In recent years, the effect of nanoparticles on human health has given rise to serious controversies. According to the Nanotechnology Consumer Product Inventory (CPI), from March 2015, the global market offered 1814 products based on nanotechnology, including 117 in the “food and beverage” category [18, 19]. In the USA, TiO_2_ NPs can be used in food if its content does not exceed 1% of the total weight of the product containing nTiO_2_ [20–22]. In Europe, the *at quantum satis* principle is in force, which means it can be used in amounts not exceeding the intended target level [23, 24].

## TiO_2_ NPs as a Food Additive

TiO2 NPs are added to many foodstuffs, including cheeses and sauces, skimmed milk, ice cream and confectionery products, e.g. as coating on sweets and chewing gum [23, 25–27]. Its content in sweets, and in particular in candy, chewing gum, chocolate and white-coated products, compared to other products, is very high, reaching 2.5 mg Ti/g of food [24, 25]. It is estimated that a child can consume even 2–4 times more TiO_2_ NPs per 1 kg of body weight (bw) a day than an adult person. In Great Britain, children under the age of 10 consume about 2–3 mg TiO_2_/kg BW/day, while adults consume about 1 mg TiO_2_/kg bw/day [6].

## Safety of TiO_2_ NPs in Foodstuffs

The wide applications of TiO_2_ NPs in the food industry give rise to many controversies regarding safety. The International Agency for Research on Cancer (IARC) classified the TiO_2_ NPs pigment as a potential carcinogenic factor from group 2B (probably carcinogenic to humans) based on mechanisms and tests involving animals regarding exposure by inhalation [28–30]. The European Food Safety Authority (EFSA) in its latest study on the safety of E171 (titanium dioxide) in 2016 found that data concerning values and exposure of humans to TiO_2_ NPs in food do not raise concerns [24]. However, with regard to the insufficiency of research data, the admissible daily intake of TiO_2_ NPs was not determined. Based on the results of tests involving animals, a safety margin of 2.25 mg TiO_2_ NPs/kg bw/day was established [24].

## Ways of Exposure and Accumulation of TiO_2_ NPs in Human/Animal Body

The effect of TiO_2_ NPs on the human body has been explored for many years. Both its exposure and toxicity to a human body/animal body has been widely investigated and discussed. The crystalline structure, particle size and coating can affect the surface charge, sedimentation, aggregation and thus toxicity of TiO_2_ NPs [31–34].

The previous in vitro and in vivo tests confirm the toxic effects of TiO_2_ NPs on human body such as altered cell cycle, constriction of nuclear membranes and apoptosis [35–38]. Studies also showed that TiO_2_ NPs can cause DNA damage [32, 39, 40] and interact with the epithelium of the small intestine responsible for absorption of nutrients. After exposure to TiO_2_ NPs by *various* ways, mainly by inhalation, injection, skin contact and absorption in the alimentary tract, TiO_2_ NPs can be found in different internal organs. In vivo tests revealed that after inhalation or oral exposure, TiO_2_ NPs accumulate in, among other places, the lungs, alimentary tract, liver, heart, spleen, kidneys and cardiac muscle. In addition, they disturb glucose and lipid homeostasis in mice and rats [41–43, 34]. Age can also be a factor playing a significant role in the harmful effect of TiO_2_ NPs. As indicated by the results of tests on young and adult rats [44], different age groups can require different biomarkers for detecting and monitoring oral toxicity of nanoparticles. In young rats, liver swelling was observed, along with cardiac injuries and non-allergic activation of mast cells in gastric tissue. On the other hand, adult animals showed insignificant liver and renal damage as well as reduced intestinal permeability and molybdenum content following exposure to TiO_2_ NPs. Wang et al. [45] demonstrated that the size of nanoparticles can affect both toxicity and accumulation of TiO2 NPs in different organs. Those authors, after a one-time oral administration of TiO_2_ NPs to mice of different size, demonstrated that larger particles (80 nm) are mainly accumulated in the liver, whereas smaller particles (25 nm) can be found in the spleen and, to a lesser extent, in the kidneys and lungs.

## Biodistribution and Absorption of TiO_2_ NPs in Human/Animal Body

All nanomaterials can differ considerably in composition, charge, morphology, specific surface area and state of matter, which has an influence on what happens to them in the alimentary tract and potential toxicity [46]. Food differs greatly in terms of its composition, appearance, structure and physical properties, which can lead to perceptible changes in the properties of nanoparticles, including their release, transport, solubility, state of matter and absorption. The nature and type of food containing inorganic nanoparticles can affect what happens to them in the alimentary tract [46, 47]. The content of water can affect the release of nanoparticles from the food matrix, whereas processing of food before it is swallowed can significantly alter the structure and properties of proteins [47, 48]. NPs, before they are absorbed in the human body, must pass through the gastrointestinal tract (GIT) regions (Fig. 2), which can alter their properties and change their potential toxicity [47]. The degree of intake and absorption of TiO_2_ NPs from the GIT into the blood circulation system can depend on many factors, i.e. species, type of particles/nanoparticles, their size, dispersability or particle charging [49, 50].

The current data is inconsistent and most of it indicates that when swallowed, most particles are not absorbed into the blood circulation system but are excreted with the GIT [49]. The results of recent studies showed that TiO_2_NPs were scarcely captured from the GIT and transferred into systemic circulation in rats and humans [51–53]. Studies concerning toxicity after oral administration to rats show a low level of toxicity at NOAEL > 1000 mg/kg bw/24 h (NOAEL—no observable adverse effect level) [49]. Cho et al. [53] demonstrated that the concentration of titanium in blood, when TiO_2_ NPs were administered to rats as particles every day (up to 1042 mg/kg bw/day), was not significantly higher than in the control group. Similarly, MacNicoll et al. [54] in their studies involving rats showed that a dose of 5 mg TiO_2_ NPs/kg body weight did not lead to considerable capturing of TiO_2_ NPs (measured as titanium) from the GIT into the blood, urine or different internal organs. Also, studies involving humans found that the absorption from the GIT into blood and urine was scarce [51]. It was demonstrated that both after administration of a single dose to volunteers (5 mg/kg bw/day) and of TiO_2_ NPs with different particle sizes, 15 nm (nanoshell), 100 nm (nanoshell) and < 5000 nm (pigments), TiO_2_ NPs had no impact on increased absorption of titanium depending on the particle size [51].

## Alimentary Tract

Studies have shown that nanoparticles can disturb digestion and absorption of food components, which can lead to deficiencies of macro- and microelements in the body [47]. Chen et al. [55] studied the toxicity of TiO_2_ NPs in mice in vivo. Different doses (0, 324, 648, 972, 1296, 1944, 2592 mg/kg) were injected into their mouth at different time intervals (24 h, 48 h, 7 days and 14 days). Mice showed strong symptoms of toxicity (loss of appetite, passive behaviour, trembling and lethargy). The highest accumulation of TiO_2_ NPs was found in the spleen that also sustained damage. Other observations included necrosis of liver cells and apoptosis, liver fibrosis and swelling of renal glomeruli. TiO_2_ NPs were also deposited in the lungs where blood clots were found that could have resulted from blockage of blood vessels. Duan et al. [56] demonstrated weight loss in mice after intragastric administration of TiO_2_ NPs anatase in doses of 125 and 250 mg/kg. This should be explained by the reduced number of intestinal villi and the resulting loss of surface of the small intestine capable of absorbing nutrients, which consequently leads to malnutrition and weight loss. Amedollia et al. [57] in their studies also showed that after oral exposure of rats to a dose of 2 mg/kg body weight, TiO_2_ NPs are capable of penetrating the intestinal mucosa. Brun et al. [58] argue that it is likely that TiO_2_ NPs would be translocated both through the ileac epithelium and through Peyer’s patches, which would lead to damage and most likely chronic failure of the intestinal epithelium. Nogueira et al. [59] found an inflammation in the small intestine of mice after they were administered TiO_2_ NPs (66 nm) by oral gavage in the dose of 100 mg/kg over 10 days.

The results of the abovementioned in vivo tests confirmed the results of in vitro studies on human intestine cell cultures. The authors [60] came to similar conclusions. Their studies on Caco-2/HT29-MTX cells showed that exposure to TiO_2_ NPs had a considerable impact on the transportation of nutrients, that is, Fe and Zn, capturing of fatty acids and IAP activity (inhibitor of apoptosis protein). They found a decrease in the number of microvilli resulting in a reduction of the surface area available for absorption of nutrients. Faust et al. [21], in their in vitro studies of the human intestine model, showed that after exposure to TiO_2_ NPs, the villi in the small intestine were destroyed. They also found that about 42% of intestinal microvilli were lost when 350 ng of TiO_2_ NPs were added per 1 cm^2^ of the medium.

The present studies provide evidence that TiO_2_ NPs have both a positive and negative effect on the quality of intestinal villi, which seems to be dependent on the dose and on the age of the animals. In young animals, the permeability of the intestinal wall is probably higher, and thus the rate of absorption and bioavailability is increased, which leads to increased exposure to TiO_2_ NPs [61].

Wang et al. [62], after intragastric administration of TiO_2_ NPs to mice over 30 days in three doses (5, 50 and 150 mg/kg bw), observed insignificant damage to the cells of the spleen (denser and larger lymph follicles in splenic tissue) in animals exposed to the lowest dose, which was not the case with higher concentrations. TiO_2_ NPs caused a significant increase in the accumulation of reactive oxygen species in the spleen of mice due to lipid peroxidation. Mohamed [63] administered 5, 50 and 500 mg TiO_2_ NPs/kg bw to mice orally and found that, even when the dose was low, TiO_2_ NPs were permanently accumulated in mice, which led to inflammation, apoptosis and oxidative stress, and consequently induced chronic gastritis.

In most of the existing studies, liver failure was observed in mice and rats exposed to TiO2 NPs. Bu et al. [64], after oral administration of TiO_2_ NPs to rats, observed liver and heart damage as the consequences of disturbances in energy and amino acid metabolism and in intestinal microflora. Duan et al. [56] came to similar conclusions. They observed an increase in liver ratios and histopathological changes in the liver after oral administration of TiO_2_ NPs (5 nm) to mice at 62.5, 125 and 250 mg/kg bw over 30 days.

In three publications, the researchers [Kreyling et al. 65–67] investigated biokinetics and translocation of TiO_2_ NPs administered via three classical ways (intravenous injection (40–400 mg/kg), oral administration (30–80 mg/kg) and injection into the trachea (40–240 mg/kg) in identical laboratory conditions. Female rats were administered single doses of anatase nanoparticles (TiO_2_ NPs) 48V isotope labelled to ensure precise tracking of translocation kinetics and total biodistribution of 48V-nTiO_2_ NPs in different tissues over 28 test days. It was found that a single dose administered orally was 99.7% excreted with faeces whereas 0.3% remained in the body for at least 7 days and continued to accumulate in the liver and spleen according to previous studies [68, 44, 45]. Other results of tests on mice show that oral administration of 5 mg TiO_2_ NPs/kg bw (5 days, 10 weeks) can contribute to intensification of an existing intestinal cancer (colon cancer) [69].

Literature also recounts cases where TiO_2_ NPs had no toxic effect. In the 90-day-long study on oral toxicity, male and female rats were exposed to TiO_2_ NPs via gastric tube at 0, 100, 300 and 1000 mg/kg bw/day. No deaths were recorded in connection with TiO_2_ NPs and no clinical, ophthalmological or neurobehavioural changes were observed due to exposure to TiO_2_ NPs. In addition, no adverse effects on body weight were recorded. The largest examined dose, i.e. 1000 mg/kg BW/day, did not cause any changes in male and female rats [49].

Similarly, Warheit et al. [70] did not find any changes in the body weight of rats after administration of 5 g TiO_2_ NPs/kg BW. Other authors [57] studying HT-29 cell cultures did not find TiO_2_ NPs to have a cytotoxic effect at different concentrations (1–20 mg/cm^2^) after 6, 24 and 48 h exposure, either. Table 1 summarises the effect of TiO_2_ NPs exposure.

**Table 1:** The effect of exposure to NPsTiO_2_ on the alimentary tract

Model	Type NPsTiO_2_	Dose	Exposure time	Effect	References
Mice	Anatase-TiO_2_	0.324, 648, 972, 1296, 1944, 2592 mg/kg	24 h, 48 h, 7, 14 days	Spleen damage, necrosis of liver cells and apoptosis, liver fibrosis, swelling of renal glomeruli	[55]
Rats	Anatase-TiO_2_	2 mg/kg	5 days		[57]
Caco-2/HT29-MTX	Unknown	106 s/cm^2^ (low), 108/cm^2^ (medium) and 1010/cm^2^ (high)	4 h, 5 days	Decrease in the number of microvilli resulting in a reduction of the surface area available for absorbtion of nutrients	[60]
Caco-2BBe1	Food grade TiO_2_, isolated from candy	350 ng TiO_2_ NPs/cm^2^ medium	19–21 days		[21]
Mice	Anatase TiO_2_	5, 50 and 150 mg/kg	30 days	Insignificant damage to the cells of the spleen in animals exposed to the lowest dose. Significant increase in the accumulation of reactive oxygen species in the spleen of mice due to lipid peroxidation	[62]
Mice	Mixture of rutile and anatase	5, 50 and 500 mg/kg	24 h, 7 and 14 days	Apoptosis, oxidative stress, chronic gastritis	[63]
Rats	Unknown	0.16, 0.4 and 1 g/kg	14 days	Liver and heart damage as the consequences of disturbances in energy and amino acid metabolism and in intestinal microflora	[64]
Mice	Anatase TiO_2_	62.5, 125 and 250 mg/kg	30 days	An increase in liver ratios and histopathological changes in the liver	[56]
Mice	Food-grade TiO_2_ was from SENSIENT COLOURS	5 mg/kg	5 s	TiO_2_ NPs can contribute to intensification of an existing intestinal cancer (colon cancer)	[69]

## Cardiovascular System

Different ways of exposure to nanoparticles can have various influences on the cardiovascular system. The influence depends on the amounts, dose of exposure, mechanisms and transfer routes, duration of exposure and the target organ [20].

Some studies showed that TiO_2_ NPs could be toxic and have a negative effect on the cardiovascular system. The inflammatory response triggered by TiO_2_ NPs is deemed one of the main causes for cardiovascular system malfunction. Increased expression of inflammatory cytokines such as TNF-α, INF-g and IL- 8 in blood after intake of TiO_2_ NPs was observed by Gui et al. [71] and Trouiller et al. [72]. Chen et al. [20], in their in vivo tests on rats, set forth a hypothesis that heart damage and inflammatory response could be possible mechanisms of adverse cardiovascular activity triggered by TiO_2_ NPs. They demonstrated that a low dose of TiO_2_ NPs could lead to potential undesirable cardiovascular effects after 30 or 90 days of oral exposure. After 90 days of intravenous administration of TiO_2_ NPs (0, 2, 10, 50 mg/kg), those authors observed heart arrhythmia manifested in reduced activity of lactate dehydrogenasis (LDH), α-hydroxybutyrate dehydrogenase (alpha-HBDH) and creatine kinase (CK). After shorter exposure to TiO_2_ NPs (30 days), changes in heart rate (HR) and blood pressure (BP) could be noted. Savi et al. [73] discovered that intra-breath in vivo administration of the saline solution containing TiO_2_ NPs (2 mg/kg) increased the rate of cardiac conduction, which results in increased likelihood of developing arrhythmia. Kan et al. [74], in their studies, demonstrated that after inhalation of ultrafine titanium dioxide (UFTiO_2_), the heart rate considerably increased and the average diastolic blood pressure was higher in response to isoproterenol.

Wang [45] evaluated the toxicity of TiO_2_ NPs (25 and 80 nm) in adult mice in comparison to fine TiO_2_ NPs (155 nm). A fixed dose of 5 g/kg bw was determined according to the procedure designed by the Organisation for Economic Cooperation and Development (OECD). Changes were identified in the following biochemical parameters in blood serum: ALT/AST, LDH (alkaline phosphatase (ALT); aspartate aminotransferase (AST); lactate dehydrogenase (LDH), which suggested liver damage after exposure to TiO_2_ NPs. In addition, nephrotoxicity and pathological renal lesions could be observed in experimental groups. Rats receiving nanoparticles 25 and 80 nm in size showed a considerable change in the activity of LDH and α-HBDH in blood serum compared to the control group, which suggested cardiac muscle damage. No pathological lesions were found in the heart, lung, testicle (or ovary) and splenic tissues***.***

Bu et al. [64] observed that daily oral administration of TiO_2_ NPs (160, 400 and 1000 mg/kg) to rats over 14 days led to disturbances in energy and amino acid metabolism and in intestinal microflora. They suggested it could cause slight damage to the liver and the heart. Comparative tests of TiO_2_ NPs toxicity after 30 days of oral exposure (0, 10, 50, 200 mg/kg BW/day) to 3-week-old (adolescent) and 8-week-old (adult) rats revealed decreased activity of HBDH and CK in young rats, which points to potential damage of the cardiac muscle [44] Hong et al. [75] in their studies involving mice showed that 6 months of exposure to TiO_2_ NPs (1.25, 2.5 and 5 mg/kg) caused damage to the cardiac muscle and pneumonia, which could be a result of disturbed expression of cytokines connected with Th1 or Th2 in the heart of mice. Table 2 summarises the effect of TiO_2_ NPs exposure.

**Table 2:** The effect of exposure to NPsTiO_2_ on the cardiovascular system

Model	Type NPsTiO_2_	Dose	Exposure time	Effect	References
Rats	Anatase TiO_2_	0, 2, 10, 50 mg/kg	30 and 90 days	Heart arrhythmia manifested in reduced activity of LDH, HBDH and CK, changes in heart rate and blood pressure	[20]
Rats	Mixture of anatase and rutile	2 mg/kg	4 h	Increased the rate of cardiac conduction, arrhythmia	[73]
Rats	Rutile UFTiO_2_	Areozol 6 mg/m^3^	4 h	Increased heart rate, increased diastolic blood pressure	[74]
Mice	Unknown	5 g/kg	14 days	Considerable change in the activity of LDH and alpha-HBDH in blood serum, which suggested cardiac muscle damage. No pathological lesions were found in the heart, lung, testicle (or ovary) and splenic tissue	[45]
Rats	Unknown	160, 400 and 1000 mg/kg	14 days	Disturbances in energy and amino acid metabolism and in intestinal microflora. It could cause slight damage to the liver and the heart	[64]
Rats	Anatase TiO_2_	0, 10, 50, 200mg/kg	30 days	Decreased activity of HBDH (hydroxybutyrate dehydrogenase) and CK (creatine kinase), damage of the cardiac muscle	[44]
Mice	Anatase TiO_2_	1.25, 2.5 and 5 mg/kg	Half a year	Damage to the cardiac muscle	[75]

*CK* creatine kinase, *LDH* lactate dehydrogenase, α-*HBDH alpha-*hydroxybutyrate dehydrogenase), *HBDH* hydroxybutyrate dehydrogenase

## Nervous System. The Brain

Nanoparticles, due to their small size, are able to cross the blood-brain barrier (BBB). When inhaled, they accumulate in three different regions of the respiratory tract: the nose and pharynx, trachea and teeth and lung alveoli. From there, through sensory nerves [76, 77], they are accumulated mainly in the areas of the brain such as the olfactory bulb and the hippocampus [3, 78, 38].

In the brain, TiO_2_ NPs can cause protein oxidation, oxidative damage [3, 79, 29] and impairment of antioxidative capacity and increased production of reactive oxygen species (ROS). Other findings include shrinkage of nuclear envelopes [38], apoptosis [33], changes in the content of microelements and macroelements, i.e. copper (Cu), potassium (K) and zinc (Zn) [80], and upset the BBB [81]. According to test results, oxidative stress (OS), apoptosis and the inflammatory response are the main mechanisms underlying the neurotoxicity of metallic nanoparticles [42]. Test results show that antioxidants can reverse neurotoxicity of metallic NPs by decreasing the production of ROS, increasing the activity of antioxidative enzymes, inhibiting the inflammatory condition and reducing the share of apoptotic cells [42].

Many studies revealed a toxic effect of TiO_2_ NPs depending on the duration of exposure and the dose of NPs. Some authors [76, 82] observed this relationship in cultured murine microglia N9 cells. They found that TiO_2_ NPs could elicit apoptosis of N9 cells in vitro, and thus present a potential risk for the central nervous system (CNS). Ze et al. [83] over 90 days administered TiO_2_ NPs to mice at three doses and found that NPs could translocate and accumulate in the brain. They demonstrated that the levels of the superoxide (O2^−^), H_2_O_2_, carbonyl protein, 8-hydroxy-2′-deoxyguanosine and malondialdehyde (MDA) in the brain of mice were increased in all groups compared to the control group. In addition, changes were identified in the expression of genes associated with OS in the brain of mice. Long et al. [84] observed that TiO_2_ NPs stimulated brain microglia to produce ROS and disturbed the production of mitochondrial energy. Huerta-García et al. [85], in their studies, found that TiO_2_ NPs had a toxic effect on the glial cells (C6 and U373P) of rats and humans. Nanoparticles induced morphological changes, damage to mitochondria and increased the mitochondrial membrane potential (MMP). Other researchers [86] after exposure to TiO_2_ NPs also observed a decrease in the mitochondrial membrane potential and the levels of nicotinamide adenine dinucleotide (NADH), the mitochondrial function and the production of ROS during mitochondrial respiration in rat tissues.

Márquez-Ramírez et al. [33] evaluated the effect of TiO_2_ NPs on the glial cells of humans (U373) and rats (C6). They found that, after 96 h of exposure, TiO_2_ NPs had a toxic effect on glial cells by inducing their apoptosis, which suggests that exposure to NPs can lead to brain damage. The study by Coccini et al. [36] showed that TiO_2_ NPs had a neurotoxic effect on human brain lines SH-SY5Y and D384. Both after short-term (acute) exposure (4, 24, 48 h; 1.5–250 μg/ml) and long-term exposure (7–10 days; from 0.05 to 31 μg/ml) to TiO_2_ NPs, a toxic effect on the studied cell cultures was observed regardless of the dosage.

Wu et al. [87] investigated the cytotoxicity of TiO_2_ NPs by means of PC12 cells (cells used as a model of dopaminergic neurons in vitro for the purposes of studies on neurodegenerative diseases). They observed apoptosis and inhibited cell cycle in PC12 cells after exposure to TiO_2_ NPs. They also noticed that nanoparticles were more toxic than micrometre particles and that anatase was more toxic than rutile. Sheng et al. [88] showed that TiO_2_ NPs had a cytotoxic effect on primary hippocampal neurons in 1-day-old foetal rats. Other authors [89] found that exposure to TiO_2_ NPs at two doses (0.25, 0.5 mg/ml) over 24 h resulted in decreased cell viability, increased release of lactate dehydrogenase and apoptosis. It was also demonstrated that the rate of apoptosis of neurons varied depending on the dose. In addition, TiO_2_ NPs led to an increase in [Ca^2 +^] and a reduction in MMP. Those studies suggest that the apoptosis of hippocampal neurons triggered by TiO_2_ NPs could be associated with the mitochondria and the signalling pathway. The authors suggest that TiO_2_ NPs contributed to a considerable increase in cytotoxicity to PC12 cells by inducing microglial activation.

In their works, the authors emphasise the potential effect of NPs on neurodegenerative diseases. Hu et al. [90], using the example of embryos of zebrafish *(Danio rerio)* and PC12 cell cultures, investigated the neurotoxicity of titanium dioxide nanoparticles. They demonstrated that exposure to TiO_2_ NPs had an effect on the development of Parkinson’s disease (PD). The results indicated that exposure to TiO_2_ NPs could lead to their accumulation in the brain of zebrafish larvae. An increase in the expression of genes (PINK1, parkin, α-*syn* and UCHL1) associated with the formation of Lewy bodies was observed. In addition, a loss of dopaminergic neurons could be noted, which is one of the characteristic features of PD. Researchers [38] demonstrated that TiO_2_ NPs accumulated in the murine hippocampus, which led to apoptosis in the hippocampus and induced impairment of spatial memory in mice. Mohammadipour et al. [91] found that after pregnant rats were administered TiO_2_ NPs (100 mg/kg bw), their offspring showed decreased proliferation of hippocampal cells and impaired spatial memory. Moreover, both the Morris water maze test and the passive avoidance test revealed that exposure to TiO_2_ NPs considerably distorted the inhibition and learning ability in the offspring. Jeon [92], to enhance the understanding of the molecular mechanism at protein level, carried out a proteomic analysis of protein in the brain of mice. In 11 out of 990 analysed proteins, the level of expression changed more than twice after exposure to TiO_2_ NPs: eight proteins had higher and three lower expression after exposure to TiO_2_ NPs. Moreover, the activity of several antioxidative enzymes and acetylcholine esterase in the brain was reduced. A reduction in the activity of acetylcholine esterase can suggest an increase in cholinergic activity by raising the level of acetylcholine, which is significant for the treatment of Alzheimer’s disease [93]. Hu et al. [94] in their studies also observed that the activity of acetylcholine esterase was inhibited after intragastric administration of TiO_2_ NPs to mice (over 60 days). In addition, they demonstrated a decrease in neurobehavioural and morphological capacity and brain damage symptoms in the Y maze test on mice. They also found inhibited activity of Na (^+^)/K (^+^) - ATPase, Ca (^2+^)-ATPase, Ca (^2+^)/Mg (^2+^) - ATPase, acetylcholine esterase, impaired function of the central cholinergic system, considerable reduction in the level of monoamine neurotransmitters (norepinephrine, dopamine and its metabolite 3,4-dihydroxyphenylacetic acid, 5-hydroxytryptamine and its metabolite 5-hydroxyindoleacetic acid) and an increased content of acetylcholine, glutamate and nitric oxide. Table 3 summarises the effect of TiO_2_ NPs exposure.

**Table 3:** The effect of exposure to NPsTiO_2_ on the nervous system

Model	Type NPsTiO_2_	Dose	Exposure time	Effect	References
Mice	Anatase TiO_2_	2.5, 5 and 10 mg/kg	90 days	Neurogenic disease states in mice	[83]
Mouse, microglia BV2	Anatase TiO_2_	2.5–120 ppm	16 and 18 h	Produce ROS and disturbed the production of mitochondrial energy	[84]
Human U373 and rat C6 astrocytoma cell lines	Mixture of anatase and rutile	20 mg/cm^2^	2, 4, 6 and 24 h	Morphological changes, damage to mitochondria and increased the mitochondrial membrane potential	[85]
Lung tissue of rats	Anatase TiO_2_	10 g/mg	1 h	Decrease in the mitochondrial membrane potential and the levels of NADH, production of ROS	[86]
Human U373 and rat C6 astrocytoma cell lines	Mixture of anatase and rutile	2.5, 5, 10, 20 and 40 g/cm^2^	24, 48, 72 and 96 h	Toxic effect on glial cells by inducing their apoptosis	[33]
Human brain lines SH-SY5Y and D384	Anatase TiO_2_	1.5–250 μg/ml 0.05–31 μg/ml	4–24–48 h, 7–10 days	Mitochondrial brain lesions, membrane damage in the brain cells	[36]
PC12 cells	5, 50, 100 and 200 g/ml for anatase TiO_2_, 200 g/ml for rutile TiO_2_	25, 50, 100 and 200 l g/mL	6 and 24 h	Apoptosis and inhibited cell cycle in PC12 cells	[87]
Primary cultured hippocampal neurons	Anatase TiO_2_	5, 15 or 30 μg /ml	24 h	Increased release of lactate dehydrogenase, and apoptosis	[88]
PC12 cells	Unknown	0.25 and 0.5 mg/ml	24 h (microglia) or 24 and 48 h (PC12 cells)	Apoptosis of hippocampal neurons, increase in cytotoxicity to PC12 cells by inducing microglial activation	[89]
Zebrafish (*Danio rerio*) embryos, PC12 cells	Mixture of rutile and anatase	0, 0.1, 1, 10 μg/mL	24 h	Accumulation TiO_2_ NPs in the brain of zebrafish larvae, an increase in the expression of genes (PINK1, parkin, α-syn and UCHL1), loss of dopaminergic neurons, which is one of the characteristic features of Parkinson’s (PD)	[90]
Mice	Anatase TiO_2_	5, 10 and 50 mg/kg	60 days	Accumulation in the hippocampus, hippocampal apoptosis, induced impairment of spatial memory	[38]
Rats	Anatase TiO_2_	100 mg/kg	21 days	Decreased proliferation of hippocampal cells and impaired spatial memory	[91]
Mice	Anatase TiO_2_	0.5, 10 and 50 mg/kg	60 days	Decrease in neurobehavioural and morphological capacity and brain damage symptoms, impaired function of the central cholinergic system, considerable reduction in the level of monoamine neurotransmitters	[94]

*ROS* reactive oxygen species, *NADH* nicotinamide adenine dinucleotide

## Conclusions

Along with global economic growth, our direct or indirect exposure to metallic nanoparticles has been increasing. With regard to new properties offered by their small size, nanoparticles (NPs) are incorporated in more and more commercial products. Regular supply of TiO_2_ NPs at small doses can affect the intestinal mucosa, the brain, the heart and other internal organs, which can lead to an increased risk of developing many diseases, tumours or progress of existing cancer processes. The mechanism behind the nanotoxicity of NPs has not been discovered yet. Many studies attribute it to oxidative stress, thus nanotoxicity is still an important area for future exploration.
